# Assessing the Reliability of Relevant Tweets and Validation Using Manual and Automatic Approaches for Flood Risk Communication

**DOI:** 10.3390/ijgi9090532

**Published:** 2020-09-05

**Authors:** Xiaohui Liu, Bandana Kar, Francisco Alejandro Montiel Ishino, Chaoyang Zhang, Faustine Williams

**Affiliations:** 1 National Institute on Minority and Health Disparities, National Institutes of Health, Bethesda, MD 20814, USA; 2 National Security Sciences Directorate, Oak Ridge National Laboratory, Oak Ridge, TN 37831, USA; 3 School of Computing, University of Southern Mississippi, 118 College Drive, Hattiesburg, MS 39406, USA

**Keywords:** Twitter, data reliability, risk communication, data mining, Google Cloud Vision API

## Abstract

While Twitter has been touted as a preeminent source of up-to-date information on hazard events, the reliability of tweets is still a concern. Our previous publication extracted relevant tweets containing information about the 2013 Colorado flood event and its impacts. Using the relevant tweets, this research further examined the reliability (accuracy and trueness) of the tweets by examining the text and image content and comparing them to other publicly available data sources. Both manual identification of text information and automated (Google Cloud Vision, application programming interface (API)) extraction of images were implemented to balance accurate information verification and efficient processing time. The results showed that both the text and images contained useful information about damaged/flooded roads/streets. This information will help emergency response coordination efforts and informed allocation of resources when enough tweets contain geocoordinates or location/venue names. This research will identify reliable crowdsourced risk information to facilitate near real-time emergency response through better use of crowdsourced risk communication platforms.

## 1. Introduction

Increased frequency and severity of climate-related hazards (e.g., floods, wildfires, hurricanes, and heat weaves) and anthropogenic hazards (e.g., mass shooting, epidemics) has brought unprecedented challenges to nations and individuals worldwide [1]. Risk and crisis communication regarding disasters are paramount in helping the population prepare for and respond to extreme events by providing essential information to plan and mitigate potential damages to life and property [2,3]. The proliferation of information technology and Web 2.0 have transformed the way individuals and organizations communicate and interact with others across the globe. For instance, according to the Pew Research Center, around 30% of Americans often depend on social media and social networking sites (e.g., Facebook, Twitter, etc.) for their news or information about specific events [4]. Consequently, traditional mainstream media have adopted new strategies to expand their presence, distribute content, and engage with consumers on social media [5]. Similarly, online content created by members of the public is being consumed and shared on various social media platforms (e.g., Twitter), thereby enriching and challenging traditional communication, especially during emergency management phases [6]. From the socio-psychological perspective, reasons that generally drive people to share information on social media are self-efficacy, self-fulfilment, altruism, social engagement, reciprocity, and reputation [7,8]. As a result, social media platforms have been used during disasters to issue warnings to the public, report damages, engage with stakeholders, and help organize relief efforts [9–13].

Citizen science-based platforms (e.g., iCoast, Tweet Earthquake Dispatch, CitizenScience.gov) allow citizens to collaborate with scientists in collecting and analyzing data, reporting observations and disseminating results about scientific problems [14]. Crowdsourcing platforms, such as Twitter and Facebook, are social media and social networking sites that allow non-experts to generate new knowledge and datasets [15,16]. Although both citizen science and crowdsourcing engage socio-culturally diverse and geographically dispersed citizens in data and knowledge creation/collection, each has subtle differences [17,18]. While crowdsourcing remains an ill-defined approach that uses large networks of people, citizen science solely uses scientists, volunteers, and lay people with interests and knowledge about a specific topic [19]. Because tweets are generated via crowdsourcing and tend to contain rumors and hoaxes, we assumed the tweets to be inaccurate and implemented a hierarchical approach to verify the reliability and relevance of the tweets using scientifically derived and confirmed data.

Despite the importance of social media in risk communication, there are challenges that need to be addressed. First, information overload due to massive amounts of user-generated content can overwhelm users attempting to gain relevant information [20]. Second, crowdsourced social media data often lack metadata providing information about the creator, time, date, device used to generate data, purpose, and standard, making it less credible [21–23]. Third, the advent of robot-controlled social media accounts, commercial spam, and collective attention spam/misinformation on social media [24,25] could also impede the quality of crowdsourced data. Finally, heuristics play a significant role in deciding what or whether to share information on social media. This has become influential during complicated and unanticipated crisis situations, thereby contributing to the possibility of introducing errors and biased judgements to shared risk information [26]. These challenges are more pronounced on crowdsourcing platforms.

Even when the above issues are controlled, information relevance determines the usability of social media crisis information. Thus, evaluating relevance of social media content is critical [10], and hence it is paramount to assess the quality and trustworthiness of data to ensure the information shared is accurate and true for decision making and public consumption during a crisis. The goal of this research is to extract risk information from tweets during the 2013 Colorado flood and assess the reliability (accuracy and trueness) of this information. This was done by examining the text and image content and comparing the content to publicly available information from federal, state, and local governments and emergency management agencies.

## 2. Literature Review

Risk communication, a principal element of emergency management, is defined as “the process of exchanging information among interested parties about the nature, magnitude, significance, or control of a risk” [27]. Risk communication is of paramount importance to governments, organizations, businesses, and individuals because it provides information about potential disasters/crises, possible impacts/damages, and countermeasures. Social media-based approaches are characterized by collaborative, participatory, and multidirectional communications that allow both impacted and interested populations to share unlimited information about a hazard, irrespective of its geographic location and time [28]. For instance, social networking sites (e.g., Facebook) and short-blog services (e.g., Twitter) were extensively used during 2017 Hurricane Harvey [29], 2017 Hurricane Maria [30], 2018 California wildfires [12,13], and the COVID-19 pandemic [31,32].

Data reliability can be defined as “the accuracy and completeness of data, given the uses they are intended for” [33]. Existing research assessing reliability of crowdsourced data tends to focus on evaluating quality of content (e.g., presence of metadata [21], detection of rumors [24]), and developing machine learning algorithms or models to asses data reliability [34–36]. Citizen scientists and subject matter experts are also used in reliability validation to differentiate and justify perceived “true incidents” [37,38]. Based on this need, more researchers have adopted the Amazon Mechanical Turk to verify the effectiveness and reliability of crowdsourced data in addition to other manual identification approaches [39–43].

Despite the abundance of existing evaluation methods, some algorithm-based studies rarely incorporate potentially relevant external data sources to the research context, such as meteorological and geospatial data in flood studies [44] and digital elevation models (DEM) in earthquake or landslide studies [45]. As a result, these studies may fail to capture all the necessary information for reliability validation. Therefore, this research designed a workflow to work closely with reference documents to extract reliable risk information.

Reliability in this research refers to “accuracy of information and the extent to which the data reflects actuality.” Using this definition, a workflow was developed to assess reliability of extracted risk information from relevant tweets that were obtained for the 2013 Colorado flood event. Using the workflow, we examined the tweet text and images leveraging human intelligence and Google Cloud Vision (GCV) application programming interface (API). The relevant tweets were extracted via several data mining techniques and can be found in a previous publication [10]. GCV API allowed automatic identification of image content, labeling of images, and automatic matching online information [46,47].

## 3. Materials and Methods

### 3.1. Study Site

The 2013 Colorado flood severely affected Front Range, El Paso County, Boulder County, and part of the Denver metropolitan area. The severe flash flooding caused by days of heavy precipitation that spanned from September 9th to 18th brought considerable damages to the region. Boulder County, the study site, received 9.4 inches of precipitation on September 12th alone, which was equivalent to the county’s average annual precipitation [48]. Other counties had relatively less but increasing precipitation from September 9th until September 15th.

### 3.2. Datasets and Processing

The datasets used in this study include historical tweets, geospatial datasets corresponding to the flood event and the study site (e.g., Boulder flood extent map, Boulder street map), and reference documents including news articles and agency reports from the National Weather Service, state, and local government agencies. A discussion of the data processing steps and analytical approaches is presented below.

#### 3.2.1. Tweets of 2013 Colorado floods

Historical tweets were identified and then purchased from Twitter Inc. using two types of keywords: (1) location names (Colorado, Boulder, Front Range, El Paso County and Boulder County, Denver metro), and (2) hazard event/impacts (flash flooding, flooding, rain 2013, emergency, impact, damaged bridges and roads, damaged houses, financial losses, evacuate, and evacuation). Any tweet that contained either the location name or hazard event/impact was included in the analysis. The tweets covered a 10-day duration from September 9th to 18th and captured all flooding events. From the 1 million tweets, we extracted 5202 (0.44%) English tweets that were geo-tagged to a location in Colorado. Our previous study extracted 720 (14% of the geo-tagged tweets and 0.31% of raw tweets) relevant tweets using six different computational and spatiotemporal analytical approaches [10,49]. The relevant tweets contained considerable flooding-related information with a threshold relevance score of 1.3 [10].

#### 3.2.2. GIS Data

To understand the spatial distribution of tweets with respect to the flood-impacted area, the flood extent dataset was obtained from the City of Boulder [50]. This dataset was generated using field surveys, Digital Globe Worldview satellite imagery, and public input from Boulder crowdsourced online applications. Street network data from the City of Boulder was used to evaluate reliability of tweets with respect to damages to flooded roads and streets [51].

#### 3.2.3. National Oceanic and Atmospheric Administration (NOAA) Warning/Alert Messages

Warning/alert messages sent by the National Weather Service during the 2013 Colorado flooding event were obtained from the NOAA Weather Forecast Office at Boulder [52]. The messages contained meteorological forecasts, observations, public watches, warnings, advisories, and areas that may be impacted during the flooding event. These alert/warning messages were used as official reference information in evaluating reliability of tweets.

#### 3.2.4. Reference Documents

Damage assessment reports about the Colorado flooding event were retrieved from federal, state, and local governments and emergency management agencies from their respective websites. The documents include “situational awareness report” [53], rainfall assessment report [54], and damage assessment report [55]. These reports provided situational awareness about cause of flooding, flooding extent, and severity, as well as damages to properties and infrastructures in affected regions. Additionally, newspaper articles that validated incidents and/or facts (i.e., damage to specific roads) were also used as reference documents [56,57].

### 3.3. Analytics and Techniques

**Figure 1.**
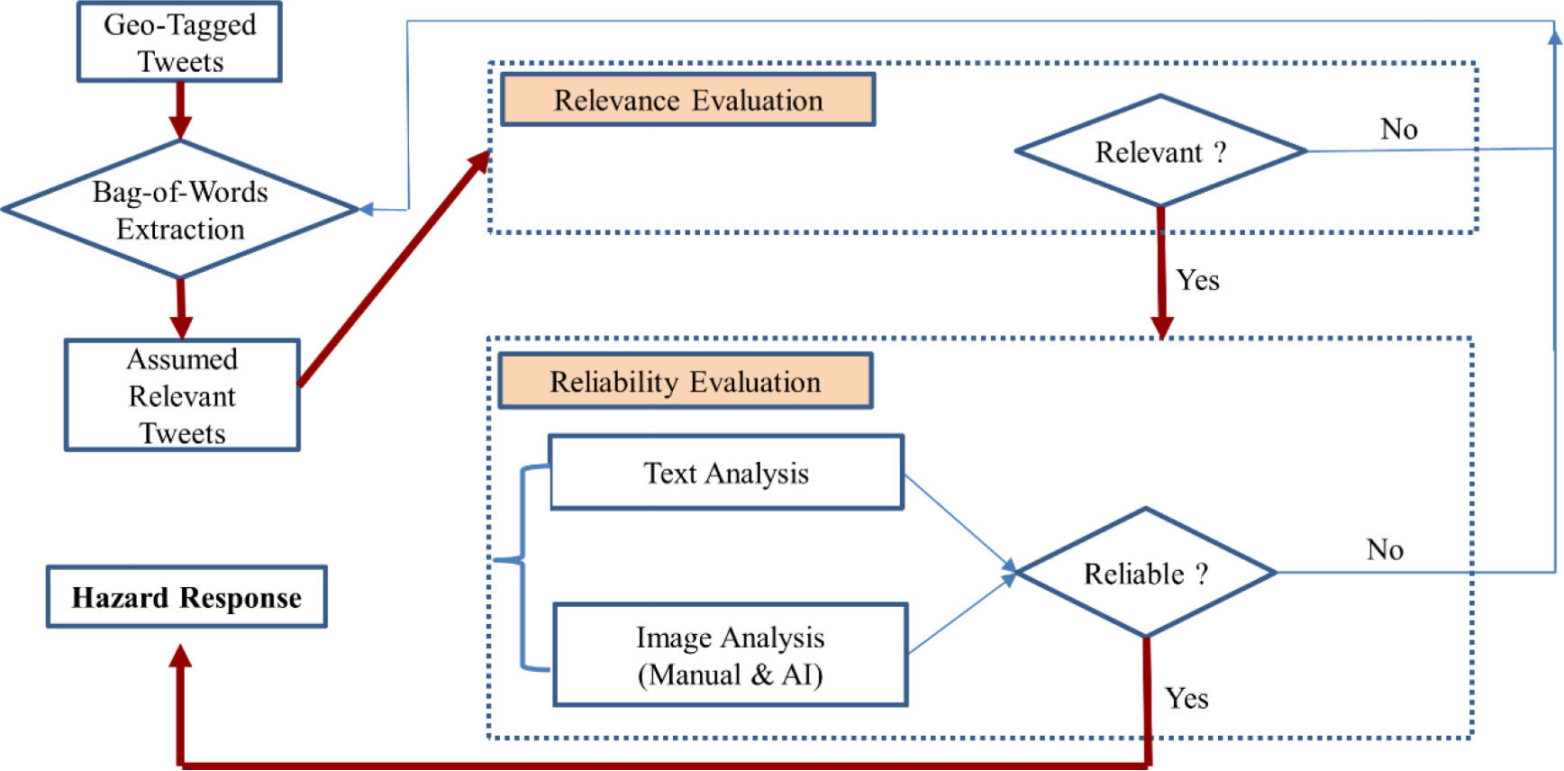
Reliability evaluation workflow.

This section presents the steps used to assess the reliability of relevant tweets. In the context of risk communication, relevant information may not be reliable, e.g., mention of the time and/or location of the event cannot be deemed as reliable unless the relevant information is verified to be accurate and true. Based on this rationale, this research sequentially extracted relevant tweets first and then evaluated their reliability (Figure 1). Specifically, the bag-of-words model was applied to geo-tagged tweets to extract assumed relevant tweets. The bag-of-words extraction used topic-specific search terms and top frequent words/hashtags to measure the relevance of a document (i.e., tweets) to the search terms and extract the assumed relevant documents. The relevance of these tweets was determined first, after which their reliability was evaluated.

The text analysis involved a few consecutive steps. The first step was to search for evident information from reference documents, especially weather warning/alert messages from the National Weather Service and the state and local emergency management agencies. Furthermore, events, names of damaged roads and streets, and the posted time of each tweet were manually identified [58] from relevant tweets and then used as keywords to search for related information in reference documents. If no such information could be found, the topic, posted time, and location of tweets was documented for further use. The next step was to holistically assess if the documented unverified tweets had any association with other tweets based on topic, posted time, or location. Finally, news information, when available, was also used as a complementary reference source. If evidence could be found from reference documents or enough tweets from multiple Twitter users presented facts that fit the hazard context in the relevant tweets, the studied tweets were considered reliable.

In the image analysis process, 308 images were downloaded from 720 relevant tweets. The images were considered reliable if they met either of the following two conditions: (1) gain evidence from credible sources, or (2) mutually prove each other. Both manual and automatic evaluation approaches were implemented to analyze the 308 images. In the manual approach, images were manually examined for roads/streets and property damage, as well as their corresponding tweet text content. Next, the image content, geographical locations, and text content were compared to reference documents. In the automatic/Artificial Intelligence (AI) approach, Google Cloud Vision API 308 images were uploaded to Google Cloud’s Vision API (application programming interface) [59], and were classified and assigned categorical labels using Google’s pre-trained machine learning models. This approach aims to leverage an existing AI (artificial intelligence) tool to improve the efficiency of extracting flood related features to facilitate the tweets reliability evaluation process.

## 4. Results and Discussion

### 4.1. Evaluation of Text Content

**Figure 2.**
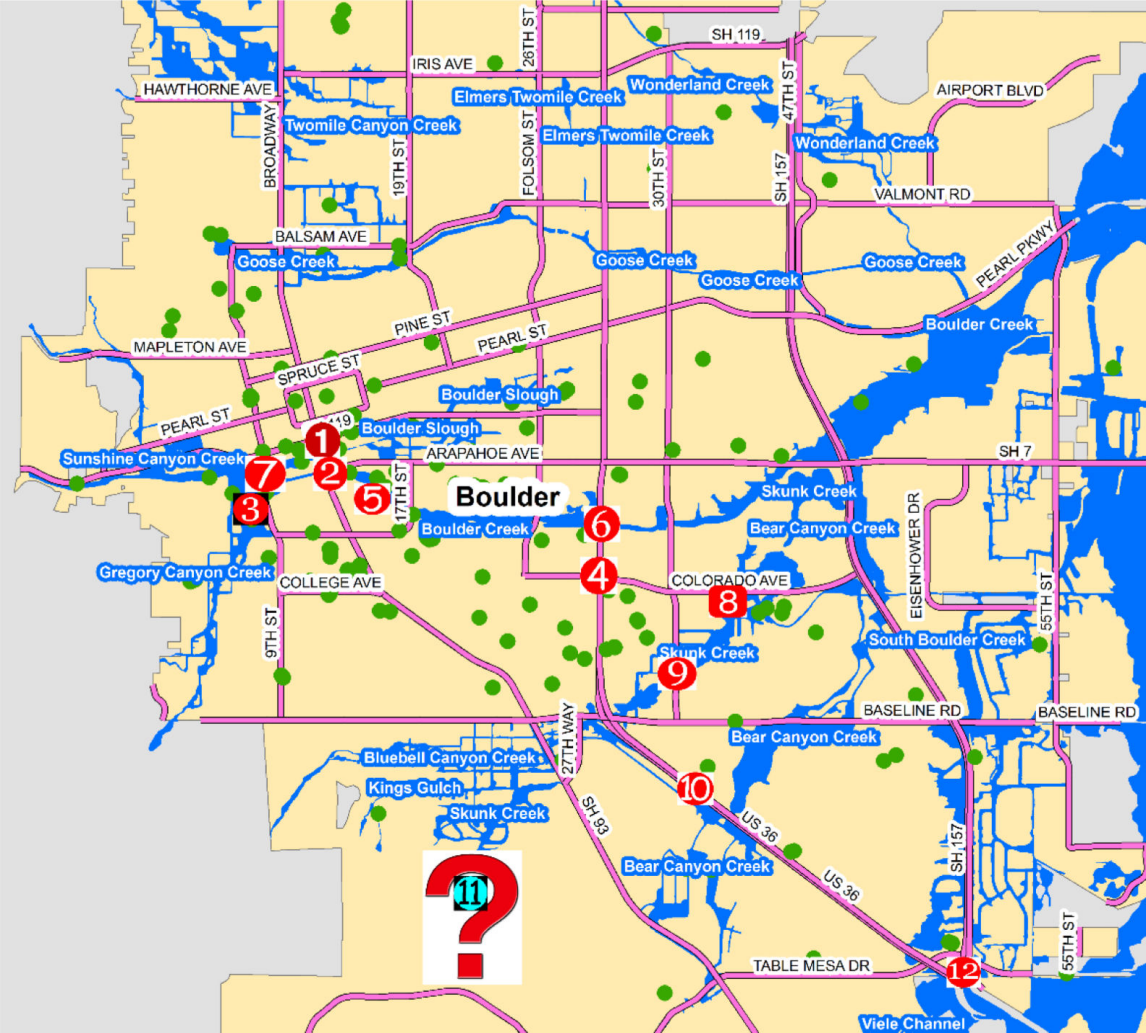
Example of identified roads/streets.

Three authors of this paper worked on manual evaluation of tweet text, and each tweet was evaluated by at least two authors to minimize human error or bias. As a result, 584 out of 720 relevant tweets were verified to have reliable information. Examples of unverified tweets include tweets solely about emotions or tweets containing information that could not be verified based on our evaluation criteria. Table 1 shows how the names of damaged roads/streets, tweet post time, and associated risk information was manually extracted. The location of the tweets shown in Table 1 were marked in Figure 2 using their ID number. A detailed description of our process to assess the reliability of each tweet is presented below.

Using the key phrase “west of Broadway,” a related NOAA warning/alert message was found from tweet #1 in Table 1: “Hourly rainfall intensity at the Sugarloaf RAWS station 6 mi. west of Boulder compared with gage height on Boulder Creek at Boulder (**west of Broadway**). The first flood peak closely followed the **heavy rainfall before midnight on September 11th–12th**, when 3.5” fell in 6 h. (Data: rainfall: RAWS via WRCC; and streamflow: Colorado DWR; plotted by Jeff Lukas, WWA)”.

The above message mentioned the gauge height on Boulder Creek at west of Broadway following the flood peak that resulted from heavy rainfall before midnight on September 11th. This corresponds to tweet #1 and explains why “Boulder Creek is about to spill its bank at west of Broadway” at 3:02 a.m. on September 12th. Therefore, tweet #1 in Table 1 was considered reliable in terms of its location, time, and content.

When searching for “Broadway” and “Arapahoe Avenue,” no direct evidence was found in tweet #2, which may be because Arapahoe Avenue is a county road and is generally too specific to be mentioned in official warning or damage assessment reports. However, as shown in symbol #2 in Figure 2, Boulder Creek flooded the crossing of Broadway and Arapahoe Avenue, allowing the observer to detect an increased water level of 2.5 feet within 10 min. Additionally, the tweet posting time (5:30 a.m.) was within the period when Boulder Creek was officially identified to have experienced a rapid accumulation of precipitation (see Section 3.1).

The crossing of 8th Street and Marine Street (symbol # 3 in Figure 2) was adjacent to and flooded by Gregory Canyon Creek, which corresponded to tweet #3 (see Table 1) indicating that the drainage at Gregory Canyon overflooded 8th Street. Based on the time, tweet #2 identified a rapid increase in water level on Boulder Creek at 5:30 a.m., and 20 min later, this tweet reported inundation of roads due to flooding of Gregory Canyon Creek, nearby Boulder Creek. This confirmed that risk information in tweet #3 is reliable based on content, time, and geographical locations.

The intersection of 28th Street and Colorado Avenue (symbol #4 in Figure 2) is between Boulder Creek and Skunk Creek, and tweet #4 was posted at the peak of the flooding when water overflowed from the creeks. The multi-day continuous rainfall flooded most tributaries and thereafter inundated most roads in Boulder City. An estimation of road damage was found in an official damage assessment report [55]: “Authorities estimate the flooding damaged or destroyed almost 485 miles of roads and 50 bridges in the impacted counties.” This tweet reported flooded roads with “knee deep water” and was posted right after continuous heavy rainfall. Therefore, it was treated as a reliable tweet.

Tweet #5 in Table 1 was posted in a similar context as tweet #4, and the user seemed to have witnessed the flooded neighborhood streets. Since this tweet was reliable, 15th Street (where the tweet was posted) could be marked as inundated so that others could avoid this road.

State Highway 36 was mentioned several times in tweets #6, #10, #11, and #12 (Table 1). The earliest mention was on September 12th when excessive rainfall continued to intensify the flooding situation. Those tweets also disclosed other details about Highway 36, such as “raining and pouring,” “flooded by over 3 feet of water,” and “its subsequent closure.” Evidence of this was also found in an official damage assessment report [55]: “Based on Federal Emergency Management Agency (FEMA) information, the flooding destroyed more than 350 homes with over 19,000 homes and commercial buildings damaged, many of which were impossible to reach except on foot. Flooding resulted in a total of 485 miles of damaged roadway, destroyed 30 state highway bridges, and severely damaged another 20 bridges. During the height of the flooding, authorities were forced to close 36 state highways. Some highways could not be repaired for weeks or even months.” These assessments confirmed the reliability of the tweets.

Tweets #6, #10, and #12 were also geo-located along Highway 36, but tweet #11 was posted beyond the city limits of Boulder. Because this tweet was posted from a place that is farther from the impacted location, it was hard to prove its reliability without referring to other tweets that also mentioned Highway 36. However, because the content mentioned in this tweet was also mentioned in other tweets, this tweet was considered reliable. Consequently, keywords that were verified to be related to important incidents/places, such as Highway 36, could be used to extract tweets that were beyond the spatial limit of the study area or that did not possess any geo-location information. This approach yields a larger volume of relevant tweets.

Tweet #7 posted at 3:22 a.m. on September 13th mentioned that a portion of 8th Street between University of Colorado Boulder and Marine Street (symbol # 7 in Figure 2) experienced severe rainfall. Given the site was located near Boulder Creek, Sunshine Canyon Creek, and Gregory Canyon Creek junction, 8th Street was highly likely to have been flooded at that time. A piece of news by Huffington Post reported that, “around 80 buildings on campus were damaged in some form, CU Boulder police tweeted, and raw sewage was flowing from a pipe in one area,” [56] confirming this tweet. A campus damage assessment report [57] also mentioned that “80 of 300 structures on the Boulder campus sustained some damage. The damage is described as ‘widespread’ but not severe.” These two news articles confirmed the reliability of the tweet.

Tweets #8 and #9 were geo-located along the flooded Skunk Creek (symbols #8 and #9 in Figure 2). While 30th Street was flooded, the adjacent Colorado Avenue was already closed. Both streets are in the Foothills area, which was reported to have been seriously impacted by flood in a damage assessment report summary: “Foothills around Boulder also saw severe flooding and debris flows” [54].

### 4.2. Evaluation of Image Content

Interactive delivery of information (stories) through images is more engaging because it is an effective way to visualize information and enables the brain to process and organize information. Through images, people can develop a deep understanding about the severity and significance of issues associated with a disaster [60]. However, previous studies have shown that around 4% of tweets are spams [61], and fake images tend to be propagated via the web, especially during crises [62]. Despite abundant research on filtering out spam or phishing tweets [63], studies focusing on diffusion of fake images are sparse [62]. Given this limitation, 308 images were downloaded from relevant tweets and two strategies were implemented to evaluate the reliability of those images. Results showed that the manual approach identified 60 (19%) reliable images compared to the AI approach which detected only 34 (11%). The following section presents the results of both approaches.

#### 4.2.1. Manual Approach

**Figure 3.**
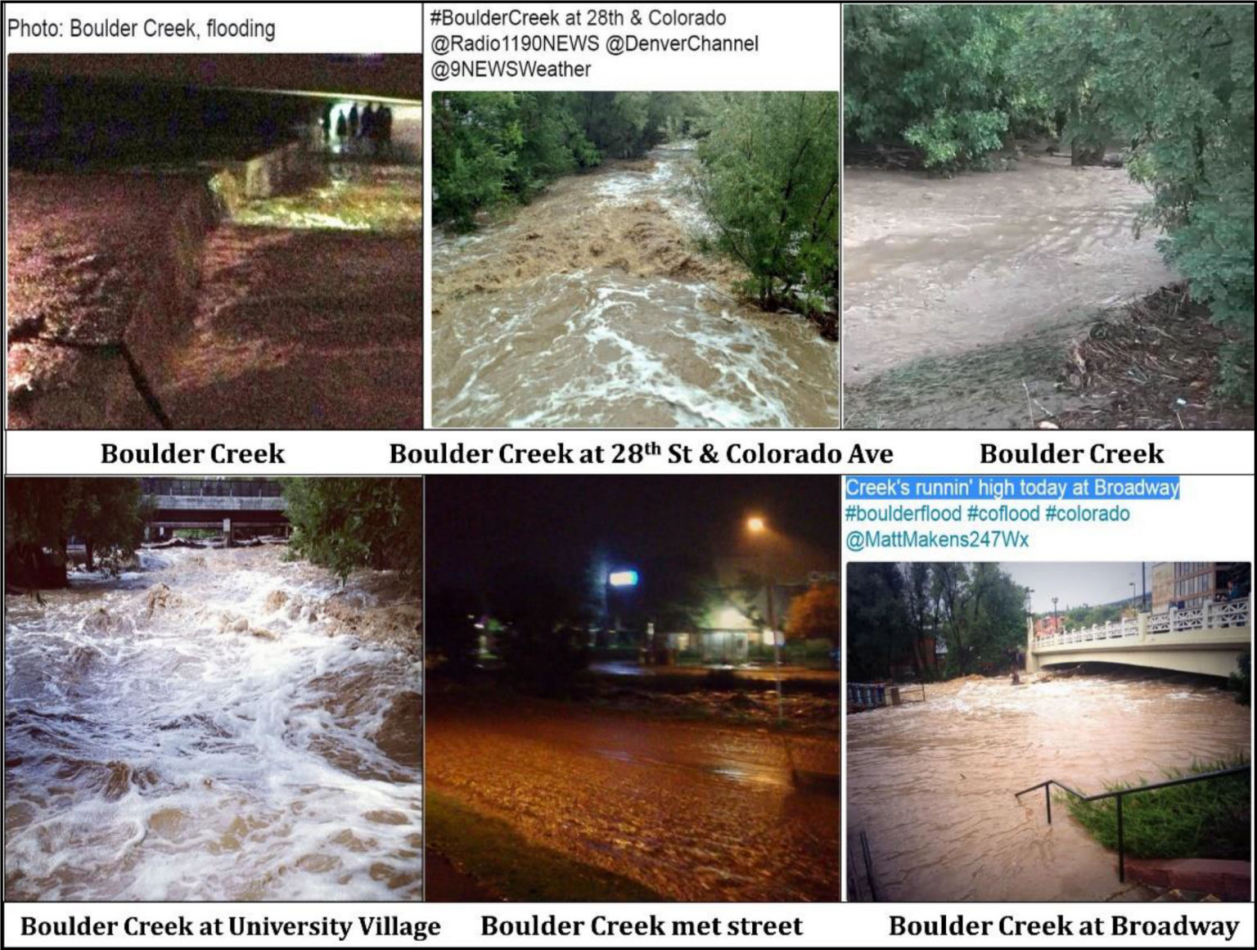
Images of Boulder Creek.

**Figure 4.**
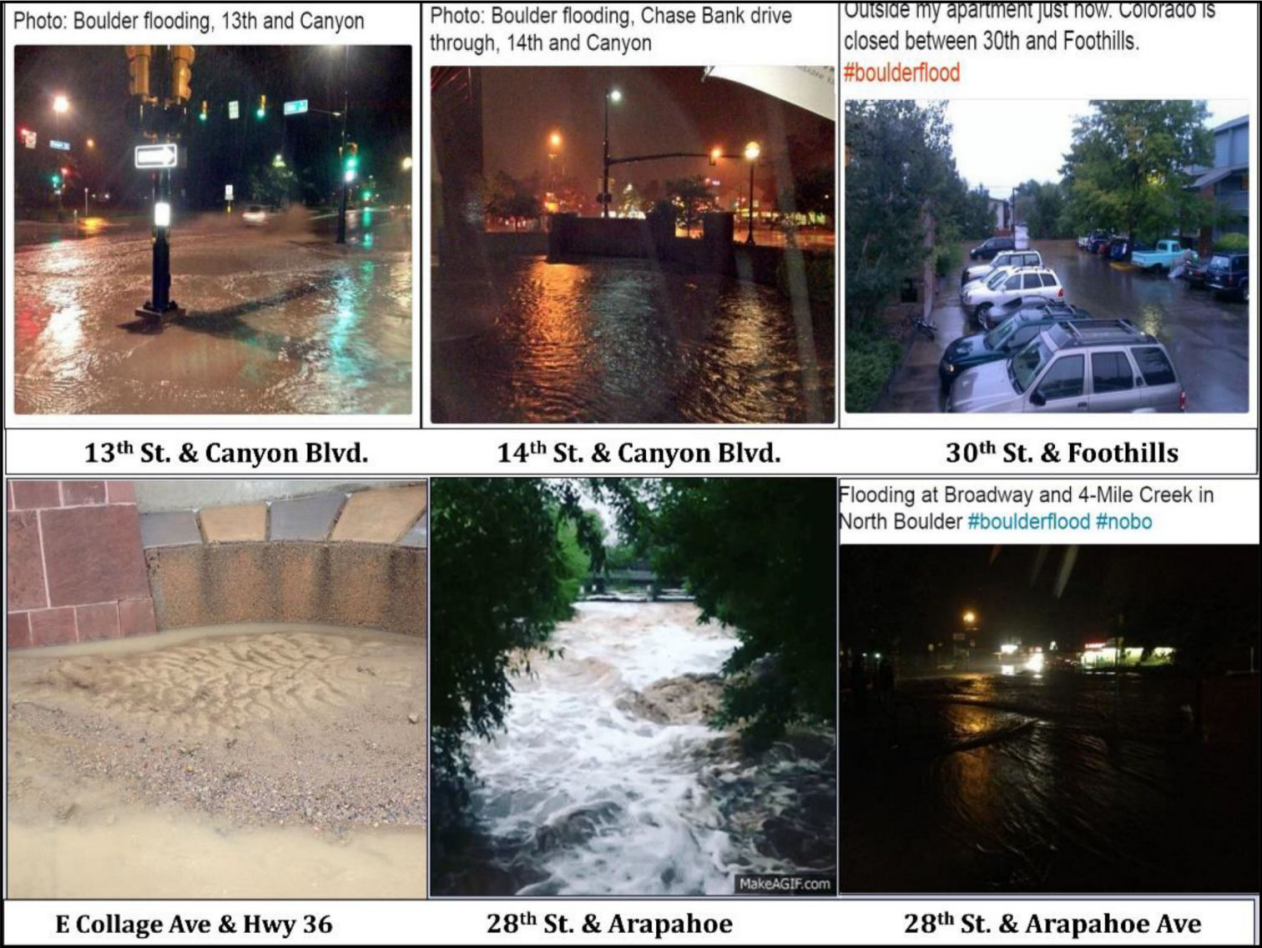
Images of flooded streets.

**Figure 5.**
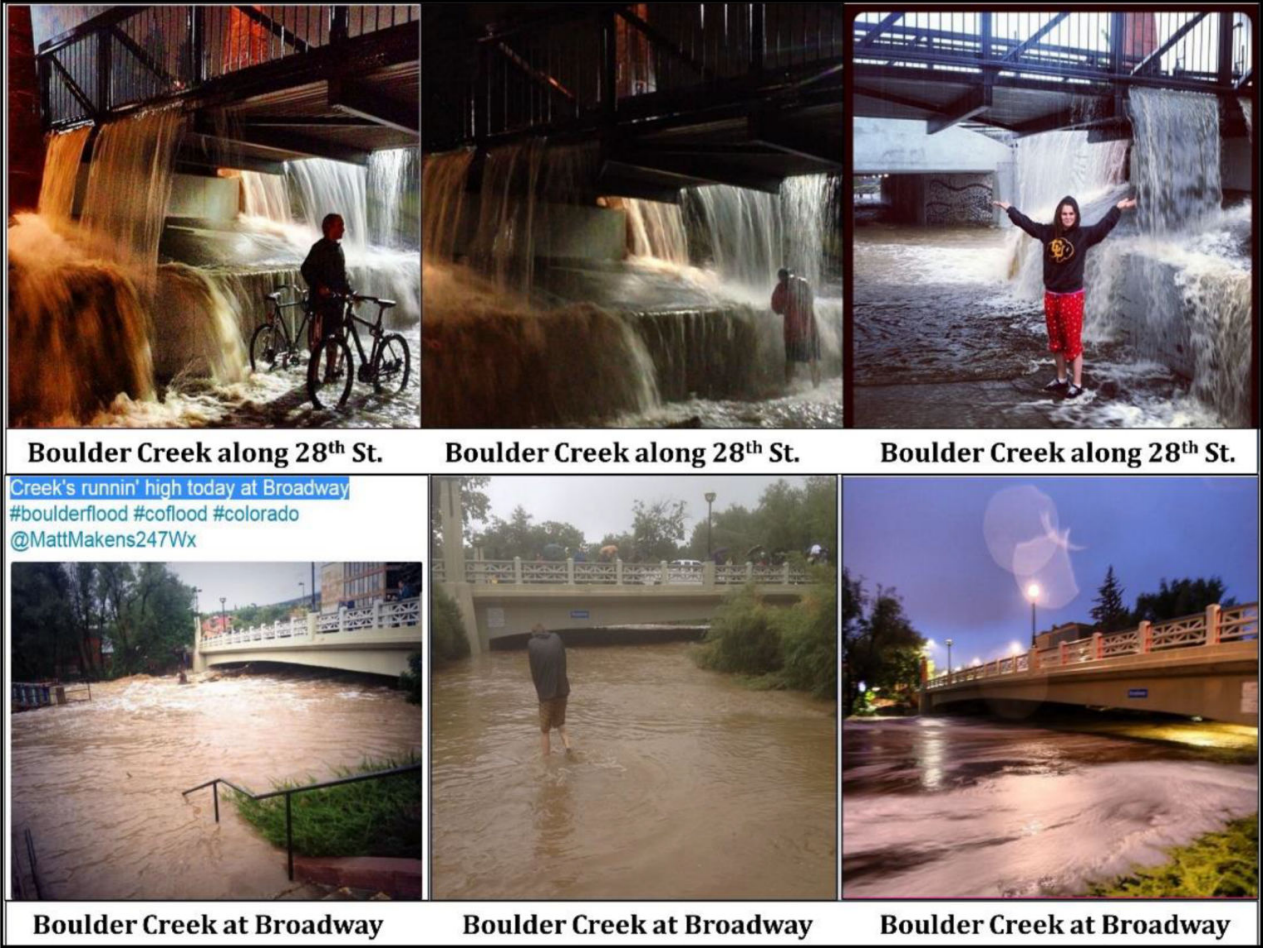
Images mutually prove each other.

**Figure 6.**
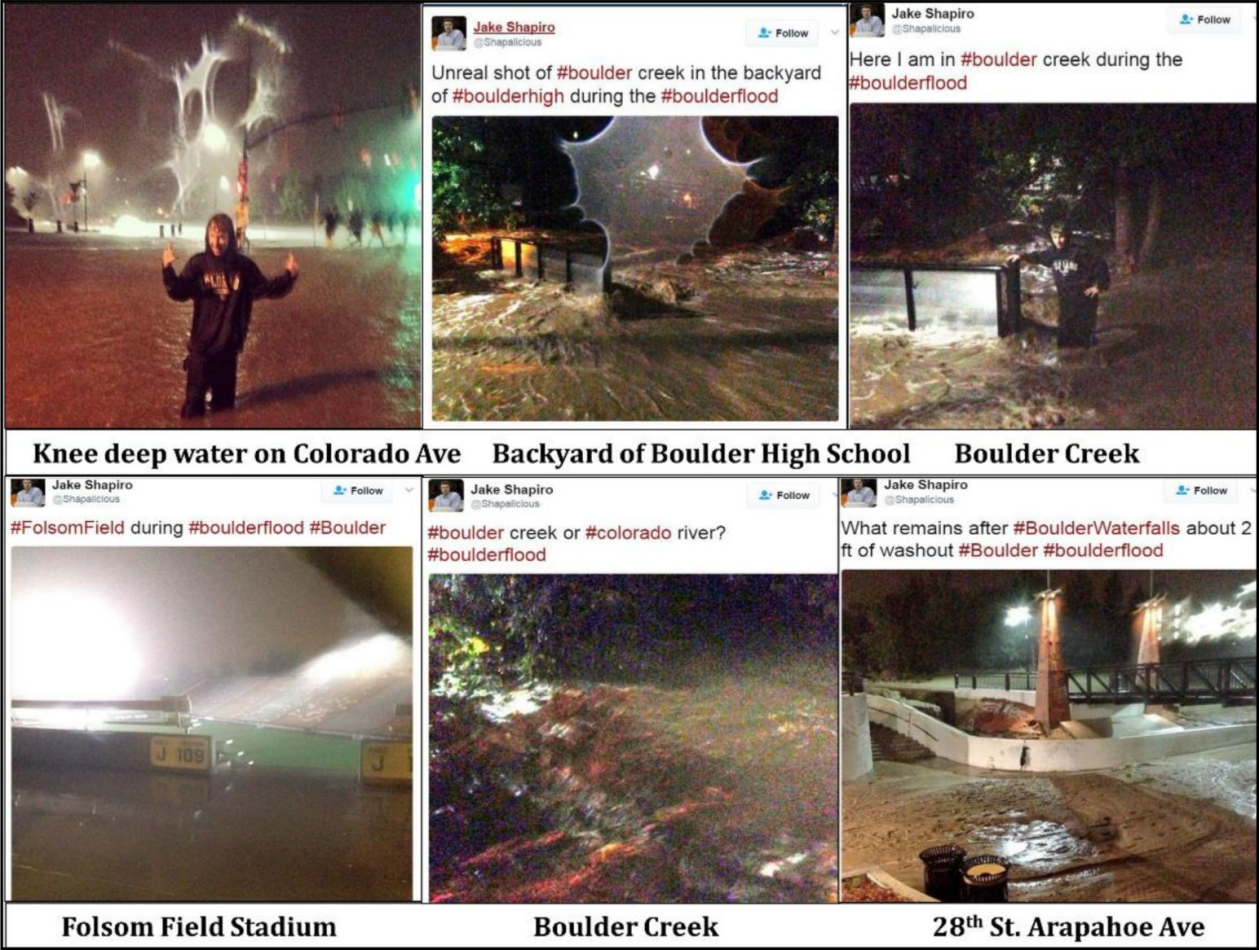
Images taken by a local news reporter.

This section illustrates the method of organizing images based on location, time, the photographer. Additionally, like-images were grouped together in order to draw comparisons and elucidate themes or topics featured in the image groups. Figures 3–5, and Figure 6 show 24 most representative images out of 308 that were identified and grouped into different themes. All images in Figure 3 depict the flood conditions of Boulder Creek at different time points, perspectives, and angles. Figure 4 includes images about submerged ground on different streets and intersections; some streets were mentioned in 4.1.2 and 4.1.4.

The images shown in Figure 5 were taken at the same location by different people, at different times, and from different angles. The flood water falling from the bridge created an unusual waterfall and attracted people to take pictures to report the severity and rarity of the flood. The bottom three images in Figure 5 recorded the increased water level at Boulder Creek under Broadway Bridge, which clearly displays the temporal change in flood severity. This finding is critical for crowdsourcing-based risk communication because massive numbers of images could mutually verify each other despite lack of external information.

Images in Figure 6 were posted by a local news reporter, who continuously reported flood situations in several locations along with pictures on Twitter. The locations that were mentioned by the reporter were: Colorado Avenue, the backyard of Boulder High School, Folsom Field Stadium, and 28th Street and Arapahoe Ave. The text and images posted by the reporter could be regarded as reliable.

#### 4.2.2. AI Approach

This section illustrates the AI approach for detecting flood-related features using GCV API and presents the result of the automatic detection. GCV API provided two types of automatic detection: image and web detection [59]. For each image fed to the API, its pre-trained machine learning models generated image detection and web detection results. Image detection results include annotated images by detecting the features of images. Web detection uses the image content and its metadata to crawl the web and detect relevant information from the internet. Accuracy of image detection is based on the availability of training data and the detection algorithm. Accuracy of web detection is based on image content, metadata, and the availability of related information on the web.

**Figure 7.**
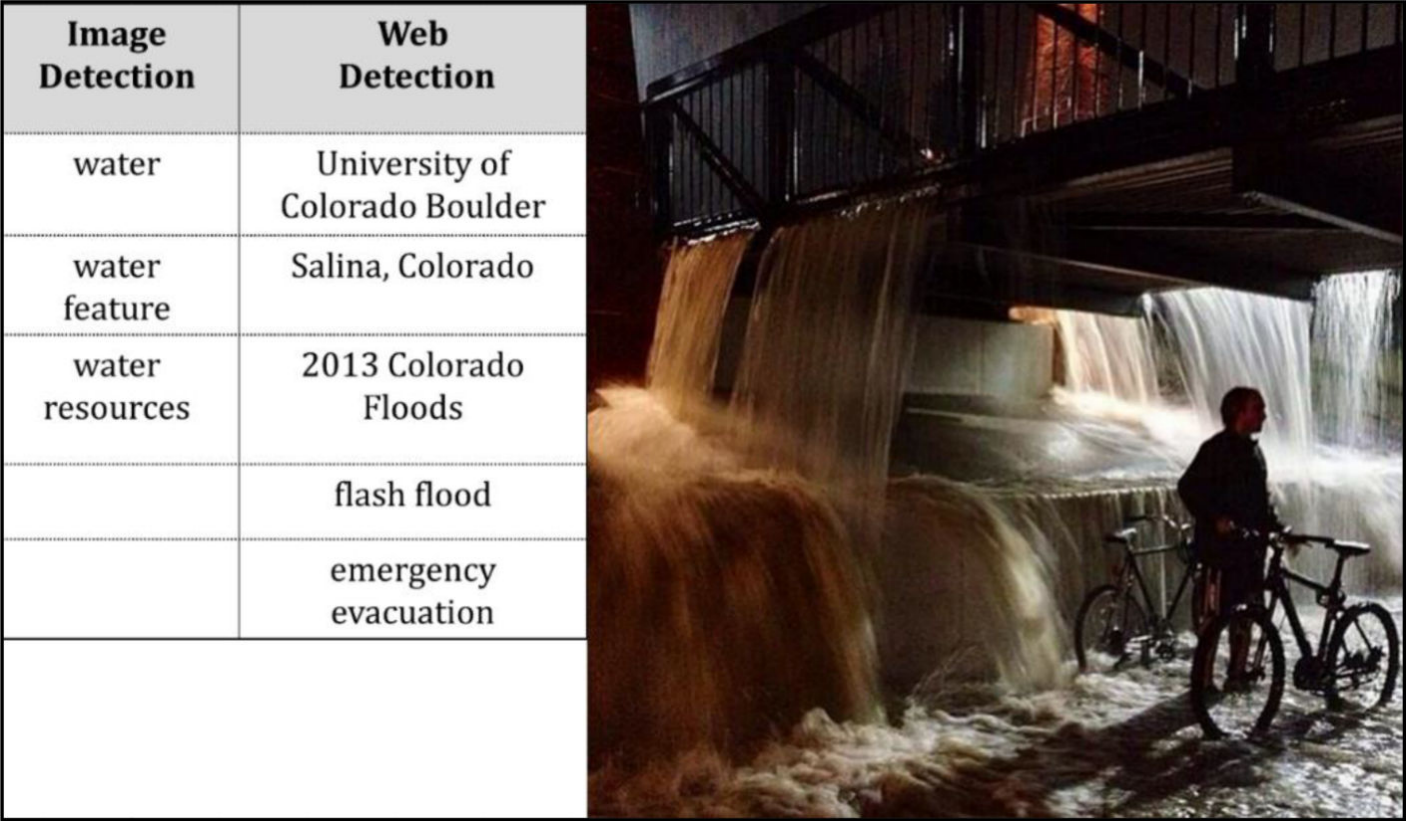
Web detection performed better.

**Figure 8.**
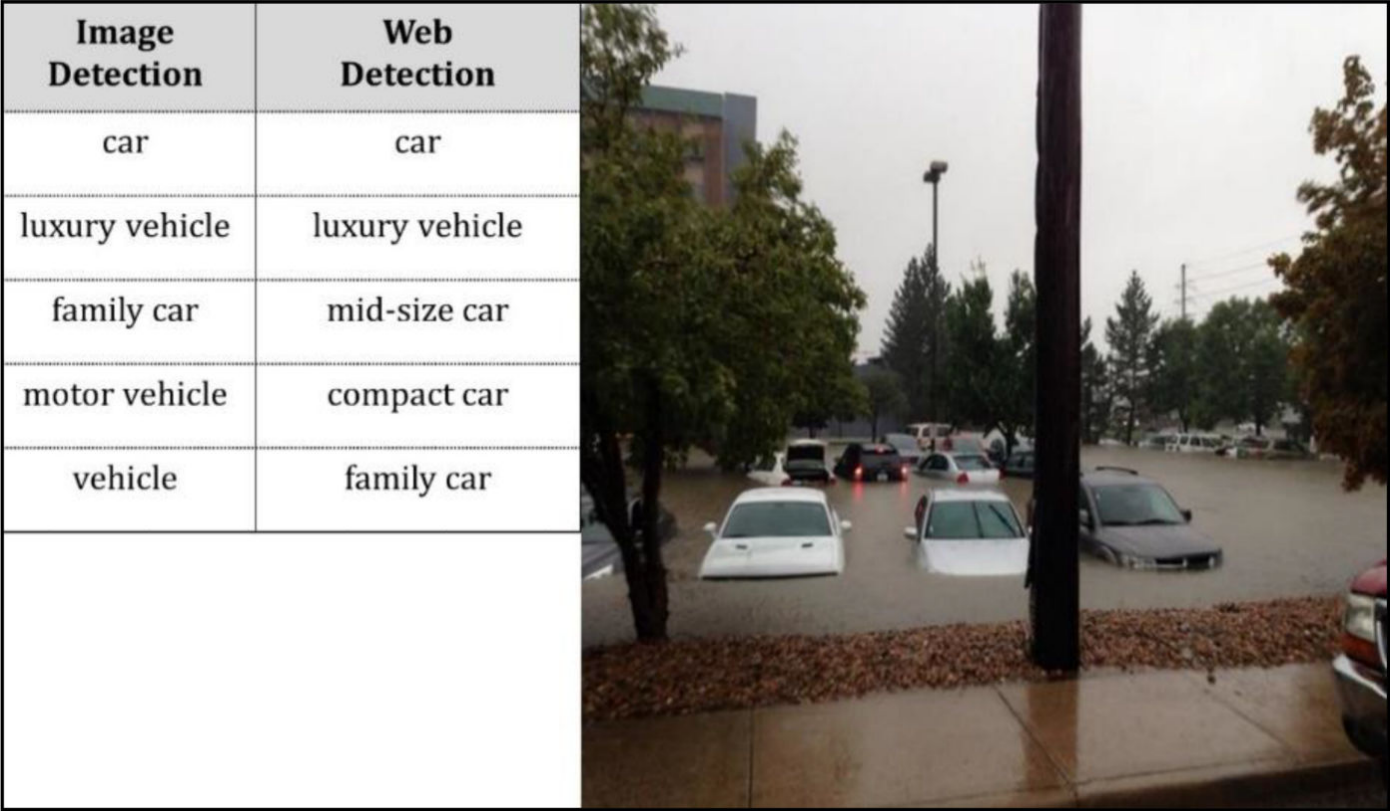
Both image and web detection were inaccurate.

Among the 34 images detected by GCV API to be relevant to 2013 Colorado flood, web detection outperformed the results from the image detection, one example of which could be found in Figure 7. According to Figure 7, web detection accurately detected the scene as the 2013 Colorado flood, while image detection only recognized the water feature in the image. On the other hand, Figure 8 shows that both modes of detection failed to identify the inundated condition of the parking lot with most cars only visible from the top. GCV API can only detect the type or size of cars, e.g., “family car” or “luxury vehicle,” largely because the sample images used to train the underlying models did not include scenes of parking lot flooding. On one hand, this suggests current limitations of AI-based image processing. On the other hand, it also implies that using images alone will cause the lack of contextual information to help us understand and make full use of the images.

### 4.3. Extracting Added Tweets Using Verified Keywords

Sections 4.1 and 4.2 identified 584 reliable tweets and 60 reliable images, which accounts for 11% and 1% of 5202 geo-tagged tweets, respectively, and 0.05% and 0.01% of all 1,195,183 purchased tweets. To make better use of this data source, we selected a group of keywords/locations (e.g., Highway 36) from the verified reliable tweets discussed in Sections 4.1 and 4.2 and used these to extract more tweets that did not possess any geolocation information. We believed that doing this would yield a larger volume of relevant tweets that were discarded due to lack of geoinformation. Despite the lack of geolocation, the time frame (September 9th to 18th, 2013) and keywords (a. location names: Colorado, Boulder, etc., and b. hazard event/impacts: flooding, rain, etc.) used to download those tweets ensured the relevance of those tweets. The keywords we used were from Table 1, which included: West of Broadway, Broadway, Arapahoe Ave, Marine St, 28th St, Colorado Ave, Boulder Creek, Highway 36/US-36, and Skunk Creek. Using these keywords, we found 2472 additional non-repetitive, relevant, and reliable tweets and 752 reliable images, which accounts for 0.2% and 0.06% of all 1,195,183 raw tweets, respectively. This is a big improvement from using geo-tagged tweets alone for this research workflow.

## 5. Discussion, Implications for Risk Communication, and Future Research

The goal of this research was to apply an integrated workflow to extract and evaluate reliable risk information to facilitate risk communication, increase situational awareness, and promote public response to natural hazards. Crowdsourced risk communication could provide valuable risk information if relevance and reliability evaluations are done properly to alleviate or eliminate data quality issues.

In this study, we implemented text and image content analysis to extract and evaluate tweet reliability. Previous research using both image and text analysis was relatively rare in Twitter-based flood research, most prior research focused on Twitter text alone. Another reason for using this approach is that information extracted from text and images is oftentimes complementary, so including both could extract more information than using either text or images.

The strengths of this research are: (1) precipitation data was used to account for the cause of flood, (2) geospatial data was used to understand the spatial extent of flooding, (3) relevant official documents were closely referenced, and (4) both manual and AI approaches were implemented in image content analysis to ensure accuracy and efficient processing time. Manual and AI approaches combined the advantages of human intelligence and computing efficiency. While leveraging human intelligence to validate textual content of tweets is not novel in Twitter text mining research, it brought a unique contribution to flood research. Specifically, it allowed identification of different scenarios and processed information beyond plain text (e.g., associate events in different images or associate events based on their proximity to events in the surrounding areas by pinpointing them on maps), which is impossible for current AI approaches to achieve. Given that the current neural networks (e.g., ResNet, UNet) used for disaster situations require human intelligence to collect and label a significant amount of training images, our manual approach complemented the AI approach. The GCV API could be replaced with other AI algorithms. However, our research workflow can be repurposed to be used by researchers interested in designing automatic or semi-automatic systems to extract reliable and relevant data and information from social media streams searching for disaster response.

Despite the advantages, both the manual and AI approaches have certain limitations in terms of their usability and implementation. First, given the time-consuming and expensive nature of the proposed manual approach, its implementation may require a team of specialists to dedicate huge efforts to extract relevant and reliable risk information in an emergency setting. One promising phenomenon to counterbalance this limitation is the emergence of volunteered citizen scientists who involve themselves in disaster response activities by voluntarily providing technical support or process information to facilitate humanitarian efforts in recent disasters [64,65]. For instance, CitizenScience.gov, citizen science efforts by the United States Geological Survey (https://www.usgs.gov/topic/citizen-science), and FEMA’s crowdsourcing and citizen science efforts have allowed citizens to participate during emergency management and response efforts to complement the activities underway by the decision-makers. With the involvement of these digital humanitarians, we believe that the workflow outlined in our research can partially or fully be adopted in disaster responses. Further, the AI approach was able to detect reliable information for 11% of the images, which is less than the percentage achieved in the manual approach (19%) and most of the images identified by AI approach were also identified by the manual approach. AI has low accuracy because it was developed for general purpose image detection and understanding, but not tailored for flood/disaster learning. If more images are used to train the AI model, it has the potential to significantly improve its accuracy. This approach requires less human labor investment, so it is complementary to the manual approach and is advantageous when a significant number of tweets are available. Finally, human errors and heuristic bias may be introduced in manual approaches, even though multiple authors cross-checked the results.

Considering the limitation of this research workflow, future research would focus on streamlining the process and automating the entire workflow of assessing relevance and reliability of Twitter data. Moreover, integration of citizen-led reliability evaluation efforts following well-informed protocols will greatly boost the usefulness of this research workflow. Space and airborne images can also be used to assess the reliability of tweets. While researchers are working to maximize the amount of risk information from Twitter, it is essential for emergency management agencies to develop easy-to-follow standards tailored for Twitter users to encourage dissemination of relevant and reliable crisis information to facilitate their use for response activities.

## 6. Conclusions

This research implemented an integrated workflow to extract and assess reliable risk information from tweets to facilitate risk communication, increase situational awareness, and promote public response to natural hazards. Both manual and automatic approaches were used to combine the advantages of human intelligence and computing efficiency. We found both the tweet text and images contained useful and reliable information about damaged/flooded roads/streets. This information could assist emergency response coordination efforts and informed allocation of resources. With the help of citizen scientists’ efforts and well-designed systems to streamline this workflow, more reliable crowdsourced risk information can be extracted to facilitate near real-time use of crowdsourced risk communication platforms during the emergency response phase.

## Figures and Tables

**Table 1.** Example of Identified Roads/Streets.

ID	Roads/streets	Posted Time	Associated Risk Information
1	West of Broadway	09/12 03:02	Boulder Creek is about to spill its bank.
2	Broadway & Arapahoe Avenue	09/12 05:30	Water at Boulder Creek has come up 2.5 feet in 10 min.
3	8th Street & Marine Street	09/12 05:52	Gregory canyon drainage overtopping the underground culvert, flowing onto 8th St. near Marine.
4	28th Street & Colorado Avenue	09/12 06:09	Knee deep water at 28th St & Colorado Ave.
5	15th Street	09/12 08:39	River taking back Boulder neighborhood street.
6	Highway 36 underpass	09/12 22:23	It’s raining! It’s pouring!
7	8th Street between University of Colorado and Marine	09/13 03:22	… basically, a raging torrent.
8	30th Street & Foothills	09/13 00:49	Colorado Avenue is closed between 30th and Foothill.
9	30th Street	09/13 01:08	Water is coming up through drains on 30th and Colorado Ave … this could get ugly.
10	Highway 36	09/13 01:30	Barely make it out of Boulder. Couldn’t get to hwy 36.
11	Highway 36	09/13 02:33	Highway 36 is flooded, not way out.
12	Highway 36 & Foothills	09/13 05:32	Over 3 feet of water flooding.
